# Is the discopathy associated with Modic changes an infectious process? Results from a prospective monocenter study

**DOI:** 10.1371/journal.pone.0221030

**Published:** 2019-08-15

**Authors:** Salim Ahmed-Yahia, Jean-Winoc Decousser, Charles Henri Flouzat-Lachaniette, Guillaume Dervin, François Roubineau, Etienne Audureau, Alexia Hourdille, Guilhem Royer, Florent Eymard, Xavier Chevalier

**Affiliations:** 1 Rheumatology Departement, University Hospital Henri Mondor, Assistance Publique-Hôpitaux de Paris, Créteil, France; 2 Department of Bacteriology and Infection Control, University Hospital Henri Mondor, Assistance Publique-Hôpitaux de Paris, Créteil, France; 3 EA 7380 Dynamyc Université Paris-Est Créteil (UPEC), Ecole nationale vétérinaire d’Alfort (EnvA), Faculté de Médecine de Créteil, Créteil, France; 4 Orthopaedic Departement, University Hospital Henri Mondor, Assistance Publique-Hôpitaux de Paris, Créteil, France; 5 Public Health Departement, University Hospital Henri Mondor, Assistance Publique-Hôpitaux de Paris, Créteil, France; Cleveland Clinic, UNITED STATES

## Abstract

**Background:**

The local infectious origin and the putative role of *Cutibacterium acnes* (CA) of a particular subtype of discopathy (Modic 1) are still debated.

**Purpose:**

To establish the association of CA in intervertebral disc (IVD) and Modic 1 discopathy in patients with low back pain.

**Methods:**

The prevalence of bacteria in IVD samples obtained by anterior approach in patient with chronic low back pain harboring Modic type 1, 2 or no Modic changes was compared to that measured in IVD samples obtained by posterior approach for sciatica. From 45 patients included in the study, 77 discs samples were obtained: 58 by anterior approach (32 Modic 1/2 changes, 26 without Modic change) and 19 by posterior approach. Conventional microbial cultures, universal 16S rRNA molecular detection and a CA specific PCR were performed.

**Results:**

12 /77 (15.6%) disc samples were culture positive. Among the 10 CA positive cultures, 5 out of 58 (8.6%) were identified from specimens obtained by anterior approach and 5/19 (26.3%) from posterior approach (p = 0.046). Moreover, the percentage of CA culture positive sample was statistically no different between the patient with or without Modic changes. The CA prevalence was lower through molecular, culture—free approaches: the universal 16S rRNA PCR was positive for 6 specimens, including one CA positive sample and the CA specific PCR was positive for one specimen obtained by posterior approach.

**Conclusions:**

In spine surgery the prevalence of CA in culture was significantly higher in IVD samples collected through a posterior approach compared to an anterior approach, suggesting a contamination process. This study did not support the CA related local infectious origin of Modic 1 discopathy.

## Introduction

Low back pain (LBP) is the 20th century disease, mentioned as the first cause of survival with handicap among 288 other diseases in a recent worldwide review [1]. Using magnetic resonance imaging, Modic et al. classified three different aspects of discopathy [2]. Modic 0 was defined as the absence of endplate changes next to discopathy. There is then a continuum in Modic changes from the type 1 through the type 3. Modic 1 changes are characterized by vertebral end plates high signal intensity on T2 weighted images suggesting local inflammation and oedema; Modic 2 changes are characterized by “fat” type redesign, high signal intensity in T1 and T2 weighted images [2–7]. The etiopathogeny of Modic 1 changes is still controversial: among the different hypothesis about their origin (local instability, biomechanically induced local inflammation, and local infection) Stirling *et al*. reported in 2001 that an infection may be the cause of the radicular inflammation [8]. Indeed, low virulent organisms as *Cutibacterium (formerly Proprionibacterium) acnes* (CA) and *Corynebacterium propinquum* were cultured from the nuclear tissue removed under strict sterile condition of 53% of patients during surgery for lumbar herniated discs [8]. Conversely, 14 discs removed from a control group of patients operated for other conditions (such as scoliosis, post traumatic spine injury, myeloma or degenerative disc disease) were sterile. Since, other studies supported these findings: the reported prevalence of bacteria in the disc and the predominance of CA as the mains bacteria varied from 19% to 71% and from 13% to 38%, respectively [9–10]. Based on this infectious hypothesis, a long-term antibiotic therapy targeting CA in LBP was tested in the context of Modic 1, inducing a significant improvement on pain and on different scales compared to placebo [11,12]. This randomized placebo controlled trial results in a high media resonance. Though, it has been highly challenged: high effect in the verum group contrasted with none response in the placebo group; none side effects in the antibiotics group; no proof of an underlying infection; and finally since this publication, none replication of those results [13].

In parallel, independent studies exploring LBP suggested that CA was a contaminant rather than a real infectious agent [14–17]. Moreover, the possibility of contamination of the disc with saprophytic bacteria originating from the skin during an epidural infiltration preceding the surgery or during surgery by posterior approach was suggested, but not proved [8]. The CA skin density on the abdominal skin being lower compared to the skin from the back, the spine surgery using an anterior approach through abdominal would be less likely to be contaminated [18]. To confirm these two hypotheses, we conducted a prospective study comparing the prevalence of bacteria (using both culture- and molecular-based methods) in disc samples collected during spine surgery from anterior and posterior approach. Then we correlated the presence of any bacteria into the disc with the Modic status.

## Materials and methods

### Setting and patients

Patients were included from the orthopaedic and neurosurgery units of a university tertiary-care French hospital (University Hospital Henri Mondor, Assistance Publique—Hôpitaux de Paris, Créteil, France) between May 2014 and March 2016.

### Inclusion criteria

We selected patients who were eighteen years old or more, whom consent was obtained and who were undergoing lumbar spine surgery by anterior approach (anterior lumbar interbody fusion (ALIF) or disc prosthesis) for chronic discogenic LBP (at least 6 months), or posterior approaches for sciatica by disc herniation. Data were collected the day before surgery: the phenotypic data (age, body mass index, tobacco and alcohol consumption, …), the level of the lumbar spinal surgery (some patients underwent surgery on multiple discs), Modic change and evidence of herniated discs on a recent MRI (number and localizations), previous spine surgery (date and level of surgery), previous lumbar epidural injection(collected as a binary response: yes or no as well as the number and the time elapsed before surgery), any previous treatments within three months preceding the surgery (non-steroidal anti-inflammatory, opiates …) and clinical data (visual analogue scale (VAS) LBP, LBP duration, VAS radicular nerve root, duration of radicular nerve root pain). We identified 2 groups:

**Group 1.** intervertebral disc (IVD) samples from patients who were undergoing lumbar spine surgery by anterior approach for disc prosthesis or ALIF, for chronic LBP (at least 6 months) with Modic type 1 or 2 or without Modic changes (Modic 0) at the lumbar level chosen for surgery.
**Group 2.** IVD samples obtained from patients who were undergoing spine surgery by posterior approach for sciatica due to lumbar herniated disc proved on MRI (control group).

### Exclusion criteria

Patients who had undergone spine surgery in the last 12 months before the inclusion or still had osteosynthesis material, patients with a cause of immunodeficiency such as chronic infection, cancer, immunosuppressive treatment, patients who had took antibiotics during at least 15 days, 3 months or less before the inclusion.

### Surgery procedure including disc collection

Since several years, our orthopaedic surgical team has implemented an anterior surgical approach to cure discogenic LBP. When patients were operated for sciatica, surgeons realized a posterior discectomy and relived a total hernia to the laboratory for microbiologic analysis. When patients underwent lumbar spine surgery by an anterior approach (an ALIF or a disc prosthesis), 2 samples of the disc were collected for microbiologic analysis: one sample from the anterior part of the disc distant from epidural space and one sample from the posterior part closed to the epidural space.

Patient received an antibiotic prophylaxis 30 min before the incision (Cefazoline, 2 g one single dose); the skin antisepsis was performed using alcoholic povidone-iodine.

To prevent contamination during the processing of the specimen, we used a sterile disposable tube including steel beads for subsequent grinding (IKA Ultra-Turrax Tube Drive, Staufen Germany). The operating nurse opened and presented aseptically the tube to the surgeons who dropped the specimen inside. The tube was then immediately sent to the laboratory.

### Microbiologic procedures

Using a biological safety cabinet, three millilitres of sterile DNA-free isotonic solution were added aseptically to the sterile disposable tube including steel beads; the biological specimen was ground for 210 seconds (50–60 Hertz speed) directly in the collection tube without additional transfer.

#### Conventional microbial cultures

The ground material was used to seed aseptically the following culture media: two chocolate agar plates incubated for 10 days and five days under anaerobic and 5% CO2 atmosphere, respectively and a Schaedler broth that was sub-cultured on anaerobic chocolate plate after 7 days or earlier if clouded. Bacterial specie identification was performed using Matrix-Assisted Laser Desorption Ionization-Time-of-Flight Mass Spectrometry (MALDI-TOF MS) and 16S rRNA sequencing.

#### Molecular detection

A commercial universal 16S rRNA gene PCR plus sequencing assay (UMD-SelectNA, Molzym, Bremen, Germany) was performed [19, 20]. This approach included (i) an automatized DNA extraction step, (ii) the human DNA destruction, (iii) the amplification and detection of the 16S and 18S rRNA genes and (iv) their identification after sequencing in comparison to dedicated bacteriologic and fungal databanks. Additionally, all clinical specimens were specifically tested for CA detection using a previously published highly sensitive specific PCR [21].

### Primary outcome

The primary outcome is to compare the prevalence of bacteria in the intervertebral disc (IVD) obtained during a lumbar spine surgery (ALIF or disc prothesis) by anterior approach (group1), to posterior approach in disc herniation (group 2), using both culture-based and molecular detection.

### Secondary outcomes

1. To compare the prevalence of bacteria in IVD obtained by an anterior approach (ALIF or disc prosthesis) in Modic 1 and 2 changes VS Modic 0.
2. To compare the prevalence of CA in the IVD in group 1 depending on the number of prior epidural injection (0 or 1 versus > 1) in patients undergoing spinal surgery by anterior approach.
3. To compare the prevalence of CA in the IVD in group 1 depending on the time between the last epidural injection and surgery (> 6 months vs < de 6 months) in patients undergoing spinal surgery by anterior approach.

### Ethics

Ethical approval was recorded from Henri Mondor Hospital n° IDR-RCB2014-A01831-44. All patients were informed that this study will not change in anyway the treatment procedure. Written informed consent was obtained from each participant.

### Statistical analysis

For categorical data, results are presented as numbers and percentages; for continuous variables, results are presented as means ±standard deviation [SD]. For positive culture rates, 95% confidence intervals were computed using the Agresti-Coull methodology. Comparisons between groups were conducted using one-way ANOVA or Kruskall-Wallis tests (3 study groups, i.e. Modic 1–2, Modic 0, Disc herniation), and t-tests or Mann-Whitney tests (2 groups, i.e. by surgical approach) for continuous data, depending on the normality of their distribution as assessed by Shapiro-Wilk test, and using Chi-square tests or Fisher’s exact tests for categorical data, as appropriate. In case of overall statistical significance between the two study groups, post-hoc tests for pairwise comparisons were conducted applying Sidak correction for test multiplicity. Unadjusted odds ratios (OR) were computed from logistic regression to quantify potential associations between study groups and the risk of positive culture, using mixed effects modelling to account for the correlation between multiple IVD samples per patient. A p-value <0.05 was considered significant. All analyses were performed using Stata 14.1 (StataCorp, Tx, USA).

## Results

**Fig 1:**
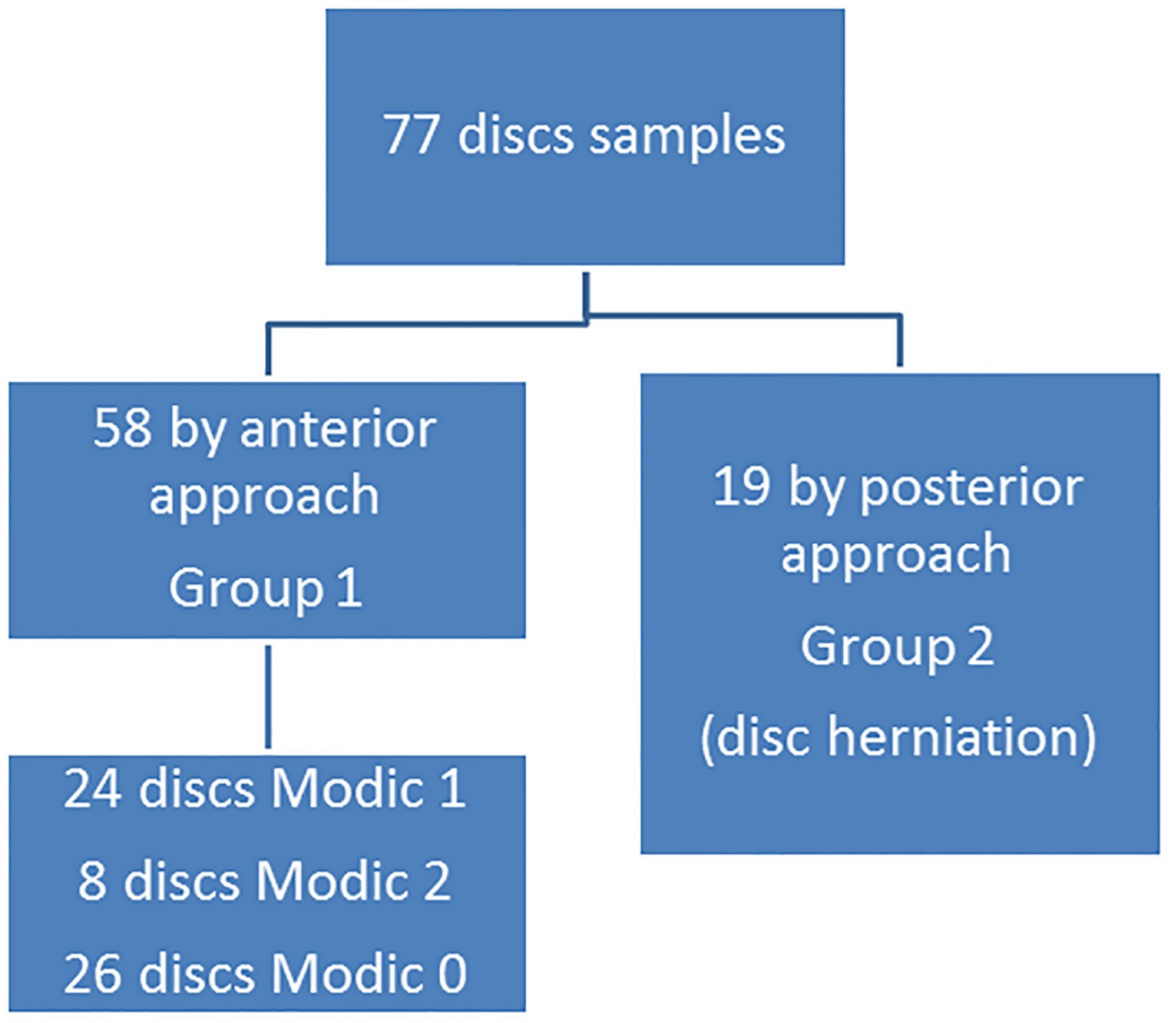
Study flow chart including the type of surgical approach and the underlying disease. From 45 patients, 77 disc samples were obtained: 58 from a anterior and 19 from a posterior approach.

The study flow chart was reported in Fig 1. Between May 2014 and March 2016, 45 patients, representing 48 IVD, were included in the study. This represented 77 discs samples, including 58 and 19 IVD parts obtained by anterior and posterior approach, respectively. Regarding Modic changes in group 1 patients, 24 IVD obtained by anterior approach had Modic 1 changes, 8 IVD had Modic 2 changes and 26 IVD had Modic 0 changes.

Patients’ characteristics were reported in Table 1. Patients operated by anterior approach were more often women (19/26 [73.1%] vs 8/19 [42.1%]; p = 0.036) and older than the group 2 (mean age was 50.4 (±10.1) in group 1 vs 41.1(±10.5) in group 2, p = 0.004). As expected the duration of LBP and nerve root pain was shorter in group 2 than in group 1. A majority of patients had a history of epidural injection with no significant difference between the groups.

**Table 1:** Patients characteristics according to study group (N = 45).

	Anterior App Group 1	Post App Group 2	p-value*
	N = 26	N = 19	
Age, years	50.4 ±10.1	41.1 ±10.5	**0.004**
Gender, females	19 (73.1%)	8 (42.1%)	**0.036**
Smokers	10(38.5%)	10 (52.6%)	0.345
Alcohol daily intake	1 (3.8%)	2 (11.8%)	0.552
Body Mass Index	26.2 ±5.4	29.1 ±6.3	0.446
LBP VAS	7.3 ±1.3	7.1 ±1.8	0.858
RNR VAS	5.8 ±2.6	6.2 ±3.0	0.527
LBP duration, months	51.7 ±56.2	19.8 ±21.1	**0.004**
RNR duration, months	30.5 ±42.2	8.3 ±7.8	**0.001**
Previous spine surgery	5 (20.8%)	4 (22.2%)	1.000
Previous epidural injection	25 (96.2%)	14 (82.4%)	0.284
Time between epidural injection and surgery, months	13.2 ±10.2	4.9 ±8.8	**<0.001**
NSAID intake	16 (61.5%)	15 (88.2%)	0.085

Results are N (%);

*p-values for global comparisons between the 2 groups

Regarding bacterial cultures, 15.6% (12/77; CI95% 9.0–25.4%) of the disc samples were positive: CA (13.0%, 10/77), *Staphylococcus epidermidis* (1.3%, 1/77), *C*. *avidum* (1.3%, 1/77) (Table 2).

**Table 2:** Microbiological results of intervertebral disc samples according to technical and to surgical approaches (N = 77).

	Total	Anterior approach fragment	Posterior approach fragment	
	N = 77	N = 58	N = 19	p-value*
Positive 16S rRNA PCR	6 (7.8%)	6 (10.3%)	0 (0.0%)	0.327
Positive CA** PCR	1 (1.3%)	0 (0.0%)	1 (5.3%)	0.253
Positive Cultures	12 (15.6%)	7 (12.1%)	5 (26.3%)	0.137
Positive CA cultures	10 (13.0%)	5 (8.6%)	5 (26.3%)	**0.046**

Results are N (%);

* p-values for global comparisons between the 2 groups

**CA: *Cutibacterium acnes*

Regarding molecular biology, 7.8% (6/77) of the 16S rRNA screening test were positive for *Staphylococcus sp*. (5.2%, 4/77), CA (1.3%, 1/77) *or Streptococcus sp*. (1.3%, 1/77). No fungal 18S rRNA was detected. The CA specific PCR detected only one positive specimen (1.3%, 1/77) obtained by posterior approach. Finally, 58 specimens (75.3%, 58/77) were negative for both the culture and molecular approaches.

### Comparing the prevalence of bacteria between the two groups (anterior vs the posterior surgical approach)

**Fig 2:**
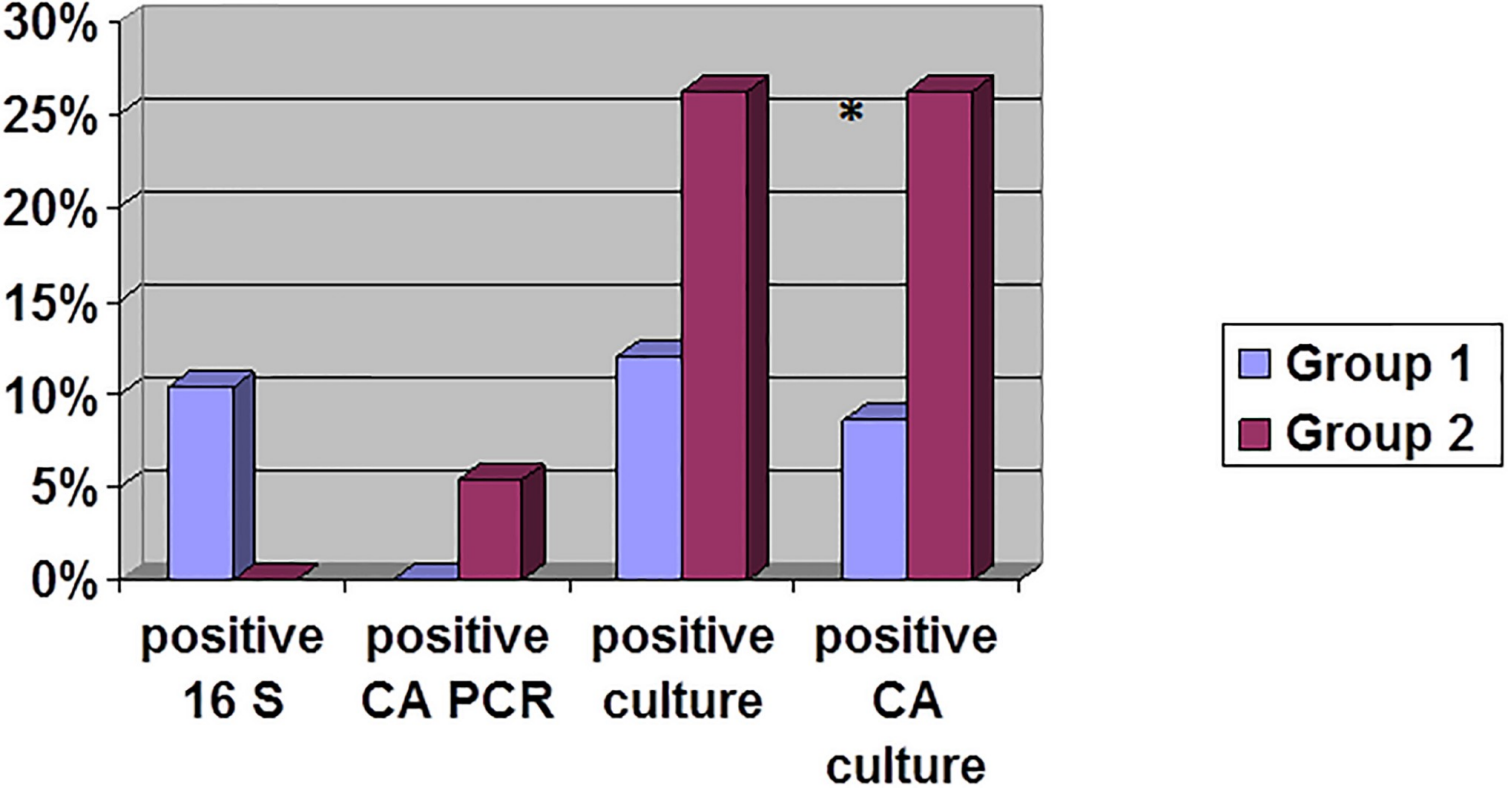
Microbiological results for intervertebral disc samples according to the surgical approach (N = 77). Microbial results included 16S and *Cutibacterium acnes* (CA) PCR detection and culture. *Group 1*: *anterior approach Group 2*: posterior approach *: *p value = 0*.*046*.

Regarding fragments obtained through the anterior approach, 14% (7/58) specimens were culture positive: CA (10%, 5/58), *S*. *epidermidis* (2%, 1/58), *C*. *avidum* (2%, 1/58) (Table 2). This rate was not significantly different in the specimens collected through the posterior approach: 26% (5/19), all positive for CA (p = 0.137). Regarding the CA positives cultures, 9% (5/58) and 26% (5/19) were identified from anterior and from posterior specimens, respectively. The fragments obtained by posterior approach were significantly more often positive than the fragments obtained by anterior approach in CA culture (p = 0.046) (Fig 2).

The 16S rRNA PCR was positive in six anterior (10%, 6/58, 4 *Staphylococcus sp*, 1 *Streptococcus sp*., 1 CA) and zero posterior fragment; the difference was not significant (p = 0.327). Considering the CA-specific PCR, all the anterior samples were negative and one posterior sample was positive with no significant difference, p = 0.253.

### Comparing the prevalence of bacteria in IVD obtained by an anterior approach (ALIF or disc prosthesis) in Modic 1 and 2 changes VS Modic 0 (Table 3)

**Table 3:** Microbiological results of intervertebral disc samples according to Modic change, N = 58.

	Total	MODIC 1–2	MODIC 0^a^	
	N = 58	N = 32	N = 26	p-value^b^
Positive 16S rRNA PCR	6 (10,3%)	2 (6.3%)	4 (15.4%)	0.393
Positive CA^c^ PCR	0 (0%)	0 (0.0%)	0 (0.0%)	1.000
Positive Cultures	7 (12.1%)	5 (15.6%)	2 (7.7%)	0.442
Positive CA cultures	5 (8.6%)	3 (9.4%)	2 (7.7%)	1.000

Results are N (%);

^a^: negative control

^b^: p-values for global comparisons between the 3 groups

^c^: CA: *Cutibacterium acnes*

A positive 16S rRNA PCR was identified in 6% (2/32) of the disc specimens obtained by anterior approach with Modic 1 and 2 changes (1 *Streptococcus sp*., 1 CA). The percentage of positive cultures in Modic 1 and 2 changes was 15.6% (5/32; CI95% 6.4–32.2%): CA (10%, 3/32), *S*. *epidermidis* (3%, 1/32), *C*. *avidum* (3%, 1/32). None of the specimen yielded a positive specific CA PCR. Only one sample was positive for CA in culture and 16s rRNA PCR in both anterior and posterior portions. There is no statistically significant difference between the 2 groups whatever the techniques used to detect bacteria: culture (p = 0.442), 16S rRNA PCR (p = 0.393) or specific CA PCR (p = 1.000).

### Influence of the total number of spinal infiltration surgery in the anterior surgical approach in the prevalence of CA culture detection

Among patients operated on anterior approach, 22 samples were collected from patients who previously benefited from 0 or 1 epidural infiltration, and 36 patients receive more than 1 epidural infiltration. The percentage of patients with a positive bacterial culture was 9% (2/22) in the group of less than 2 infiltrations and 8.3% (3/36) in the group with more than 1 epidural infiltration (p = 0.489). Thus, our results suggest that the number of infiltrations do not influence the percentage of bacterial contaminants.

### Influence of the time delay between epidural infiltration and surgery (group 1< 6 months vs group 2 > 6 months) in patients operated by anterior approach in the prevalence of bacterial detection

Regarding bacterial cultures, 1 specimen was positive in the group 1 (surgery in the 6 months) and 2 specimens were positive in the group 2 (surgery over 6 months) and 1 (OR 0.37, p = 0.23). Similarly regarding 16S rRNA detection, there was no significant difference between the 2 groups: 5% (3/56) (< 6 months) vs 8% (1/12) (> 6 months). The only 16S rRNA PCR and culture positive specimen originated from a patient who was in the infiltrated group less than 6 months before surgery. No specific CA PCR was positive among the 56 samples. In our study the prevalence of CA in culture was significantly higher in IVD samples collected though a posterior approach compared to an anterior approach, suggesting a contamination process.

## Discussion

The putative role of infectious agents in the physiopathology of Modic changes in the IVD is controversial and may have dramatic therapeutic implications. Owing to the necessary antimicrobial stewardship and to the risk of antimicrobial resistance selection and spread, all antibiotic prescriptions must be carefully justified. Our results clearly point out the presence of bacteria in the disc, and especially of CA, as a contaminant of the culture process that is not confirmed by specific PCR performed directly from the specimen.

Because of clean surgery doesn’t mean the absence of bacteria, the antimicrobial prophylaxis is the cornerstone of the preventive measures since several decades in the orthopaedic and the spinal surgery domains. Owing to the nature of the suspected bacteria that are mainly commensal species from the skin, the distinguishing between infection and contamination is of paramount importance. Interestingly, CA, the main involved bacteria, is a well-known skin commensal, opportunist pathogen but also a very common culture contaminant; when identified from a clinical specimen its clinical relevance is always questionable [18]. Indeed, numerous studies identified CA as a contaminant of blood products, tissue cultures, and surgical wounds; the significance of this widely abundant skin commensal is consequently strongly debated [22–25]. In order to sort true infective process from clinical sampling and/or laboratory-based artefacts, we performed a prospective study focusing on this issue using a bundle of dedicated protocols. Owing to the CA predominance in the back of the body, we aimed to evaluate the contamination of the specimen in performing an anterior surgical approach, collecting for the first time both the anterior and posterior fragments and compare to the specimens obtained by posterior approach for sciatica. One another strength of our study was to combine highly sensitive methods (broad range 16S rRNA PCR detection, enriched and prolonged culture) and a high specific approach (CA targeted PCR which was performed extemporaneously without any culture step). The results of the culture-based approach highlighted the extreme difficulty to prevent the specimen contamination even in multiplying the precautions: the utilization of a disposable tube to collect and to grind specimen rather than a sterile tube follow-up to re-usable porcelain mortar, manipulation of specimen in a controlled atmosphere (e.g. an operating room, a biological safety cabinet). Then a significant part of the specimens was positive for a large range of skin commensal bacteria, including the Staphylococcus, Cutibacterium and Streptococcus genus, without any statistically significant difference between the groups whatever the approach. The fragments obtained by the posterior approach were statistically significantly more frequently positive than fragments obtained by anterior approach for CA cultures. Our result confirmed the results from Rigal *et al*. who collected biopsies from 313 discs via an anterior retroperitoneal approach from patients presented Modic 1 (78%) of and Modic 2 (15%) discopathy [15]. Only 6 positive cultures were identified (1.6%), 5 in Modic 1 and 1 in a Modic 2 patients, including 2 CA. A pathological study of the disc samples showed no inflammatory changes and no real infection at the 6 months follow-up [16].

The higher percentage of positive specimen for the culture-based approach (16%) comparing to the 16s rRNA PCR method (8%) confirmed the high-sensibility of the first one, without prejudging the clinical significance of the cultured bacterial isolates. It has been established that when microbiologists tried to enhance the sensibility of their culture-based methods, for instance in increasing the duration of incubation or in performing additional treatment of the specimen as sonication, the final contribution was mitigated between true infectious agents and contaminants [26]. The determination of the best threshold to separate infectious bacteria from contaminant is still debated. Recently, the implementation of next-generation sequencing technologies confirmed that CA was the most common contaminant: in the work of Street *et al*. focusing on orthopaedic-device-related infections, CA was identified in a negative control and considered as a contaminant in 7% of the samples tested using a metagenomics approach [27]. The authors underlined the need to carefully interpret the presence of CA and to multiply samples per patient. For the first time we tested on disc samples the highly specific CA PCR published by Sfanos *et al*. and showing a sensitivity very close to the one UFC/ml sensitivity of the cultures [21]. In the first group of IVD obtained by anterior approach, no sample was positive with this PCR which, according to the protocol, was the one performed with the least human handling.

We don’t find any influence of the number of epidural injections of steroid before the surgery and the delay between surgery and steroid epidural injection, but the few numbers of patients who have not epidural injection can have skew this result.

If the infectious trail is ruled out, what could be the cause of the Modic 1 changes? The etiopathogenic mechanisms leading to Modic 1 change remain controversial but the main theory is that a biomechanical stress may impact the complex of the disc and the vertebral subchondral bone endplates. Inadequate response to mechanical stresses applied to the degenerated intervertebral disc has been suggested to contribute to disc disease activation (DDA) [28]. A genetic background and local environmental factors environmental factors such as smoking, overweight and workload may favour the disc degeneration and subsequent Modic changes [29]. As a result intervertebral disc “activation” could reflect vertebral-endplate subchondral bone microtrauma related to the loss of the intervertebral disc ability to absorb repeated shocks [30]. This active discopathy is associated with local molecular inflammation marked by release of pro inflammatory mediators, production of metalloproteases and activation of osteoclast that contribute to extracellular matrix degradation and bone remodelling [31]. On the other hand the hypothesis that systemic bone fragility may induce active discopathy has not been confirmed [32].

Our work presents some limitations. The study was monocentric and the total number of patients was only 45; our findings must be confirmed using a larger sample of patients through a dedicated prospective multicentre study. Nevertheless, the collected specimens are precious, and our sample of surgical biopsies could be considered as significant and representative of the pathology. Additional new molecular methods as 16S metagenomic or whole genome shotgun sequencing must be performed. However, as previously reported, the contamination issue could not be ruled out through this costly approach.

The strengths of our work were the dedicated and prospective nature of the study, the implementation of different methods combining microbial and molecular methods to identify the presence of bacteria, the strict aseptic collection of the disc samples, the differentiation between anterior and posterior surgery and the correlation with Modic status.

## Conclusion

Our study highlighted the presence of bacteria in specimen culture, especially CA, in peri-operative specimen collected from patients with or without Modic changes. To date our results challenge the “infectious hypothesis” of the Modic changes and support the implementation of larger studies including new microbiological technologies and more strict criteria to define disc infection, as for prosthetic joint infections. So in the meantime, the pros and the cons of long-term and extended spectrum antimicrobial therapy must be carefully weighed in this area of incertitude.
